# Psychological and Livelihood Impacts of COVID-19 on Bangladeshi Lower Income People

**DOI:** 10.1177/1010539520977304

**Published:** 2020-12-08

**Authors:** Alak Paul, Tapan Kumar Nath, Janardan Mahanta, Naznin Nahar Sultana, A. S. M. Imrul Kayes, Sharifa Jahan Noon, Md. Akib Jabed, Sumana Podder, Sujat Paul

**Affiliations:** 1 University of Chittagong, Chittagong, Bangladesh; 2 Nottingham University Malaysia Campus, Semenyih, Selangor, Malaysia; 3 Unnayan Prochesta, Tala, Satkhira, Bangladesh; 4 Premier University, Chittagong, Bangladesh; 5 Chittagong Medical College, Chittagong, Bangladesh

**Keywords:** COVID-19, mental stress, livelihood impact, lower income people, Bangladesh

## Abstract

The objective of this research is to understand the psychological and livelihood-related impacts of coronavirus disease 2019 (COVID-19) on Bangladeshi lower income group people who depend on daily earnings for their living. Following the convenience sampling method, 576 respondents were interviewed for quantitative data and 30 in-depth interviews for qualitative information in several districts of Bangladesh. To 94.1% respondents, livelihood has been affected by the COVID-19 outbreak with an overall score of 3.20 ± 0.77 on a 4-point Likert-type scale. In comparison to unemployed respondents, daily workers have been hardly affected by the COVID-19 outbreak (odds ratio [OR] = 7.957; *P* < .01), and so they are going outside more frequently in search of jobs (OR = 9.984, *P* < .01). Due to fear of COVID-19 infection and lack of livelihood means, respondents (76.6%) have been stressed out (overall score 3.19 ± 0.81 on a 4-point Likert-type scale), and those working in industries (OR = 5.818, *P* < .01), farmers (OR = 3.029, *P* < .05), and day laborers (OR = 2.651, *P* < .05) have been highly stressed.

## What We Already Know

- Coronavirus disease 2019 (COVID-19) would badly affect the livelihood of almost half of the global workforce living in urban and rural areas.
- Many low- and middle-income group of people of Bangladesh have already lost their jobs and other income sources due to the substantial effect of the COVID-19 pandemic.
- The mental health and well-being of the societies as a whole have severely been affected by COVID-19.

## What This Article Adds

- The combined effect of fear of being infected by COVID-19 and lack of confirmed support from the government has made the life of lower income group vulnerable and stressed out.
- Ignoring the fear of COVID-19 infection, a substantial number of poor people were going outside regularly during the lockdown period in search of jobs as they had no other options to maintain their livelihood.
- The impact of the COVID-19 outbreak on peoples’ livelihood was high to extreme, which indicates that the lower income people were getting more marginalized than before and becoming a member of the hardcore poor due to the pandemic.

## Introduction

The global impact of the coronavirus disease (COVID-19) pandemic is expanding daily on the poor due to job loss and other shocks to income and diminished livelihoods.^1^ The most vulnerable portion of society includes poorer households and those dependent on informal employment. It includes casual day laborers, small-scale producers, and many more who have less access to social protection because of a smaller amount of savings or limited alternative sources of income both in urban and rural settings.^2^ In rural areas, poor people are at risk of losing their prime income source as they cannot sell their agricultural products or are incapable of storing their produce or have difficulties in the process of producing new products. On the other hand, in the urban context, poor people are completely dependent on incomes from labor or self-employment. The shocks and stresses of the COVID-19 crisis worldwide are leading to devastating socioeconomic disruptions of people and both lives and livelihoods are at risk due to this pandemic.^3^ Tens of thousands of people are losing their income and falling into the trap of impending poverty, as a direct consequence of the economic crisis.

Recent statistics of the International Labour Organisation revealed that because of the coronavirus pandemic, 50% of the global workforce may lose their livelihoods, as 1.6 billion workers in the informal economy are at immediate risk of losing their income source.^4^ Furthermore, the number of people living in poverty will increase by 2% for every percentage point of global economic slowdown.^5^ Sumner et al estimated that COVID-19 could lead to an increase in global poverty for the first time since 1990.^6^ Overall, socioeconomic conditions of the mass of people in developing nations, forced lockdown without ensuring the fundamental human needs, weak governance, communication, infrastructure, and health care facilities would create public anxiety and disturbance in life.^7^ The mental health and well-being of all societies have severely been affected by this crisis and many people are distressed due to the immediate health impacts of the virus and the consequences of physical isolation, fear of losing loved ones, and fear of death from hunger.^8-11^

In Bangladesh, the first COVID-19 positive case was reported on March 8, 2020.^12^ Just a month later, the number of affected people rose to 164,^13^ and as of November 2, 2020, total number of positive cases reported was 409 252, and of them, 5941 people died in this pandemic in the country.^14^ Just within 7 months, the severe acute respiratory syndrome coronavirus 2 has infected a substantial number of citizens and the virus is still transmitting among people like a chain reaction. The ongoing pandemic will not only affect the national economy but also the financial status of millions of families in Bangladesh.^15^ Due to the lockdown, marginal people such as rickshaw-pullers, day-laborers, domestic workers, transport workers, street vendors, and construction laborers have already been experiencing jobless condition and they are the worst sufferers of this outbreak.^11^ Moreover, the lockdown hit hard low- and middle-income people who have lost their jobs and income sources that ultimately resulted in psychological anxiety, stress, and fear of death from hunger.^16,17^ The press reported that the average earnings of daily breadwinners in cities and rural areas of Bangladesh have declined by almost 80% since the coronavirus outbreak.^18^

The aim of this research was to investigate the psychological and livelihood impacts of COVID-19 on Bangladeshi lower income group people. By “lower-income group people of Bangladesh” we mean those people who depend on daily earnings for their living. These people are generally daily wage laborers, unemployed, small vendors or having informal jobs, and daily income is generally not higher than the national poverty line (US$2 per day). These people comprise about 31% of the total population in Bangladesh.^19^ We expect that the findings of this research would help relevant authorities to undertake measures for the well-being of lower income group of people in this pandemic.

## Methodology

### Sample Population and Data Collection

This study was conducted across Bangladesh targeting a lower income group of people (mostly daily income earners) in suburban areas. Because these fractions of people usually have limited access to the Internet, we opted to conduct physical interviews maintaining at least 2 m distance. We followed convenience sampling to select respondents because this is the most common nonprobability sampling strategy where respondents are selected in an ad hoc fashion based on their accessibility. We employed a number of field assistants for data collection and briefed them about interview protocol (e.g. safe distancing, wearing masks, etc).

This study used both quantitative and qualitative approaches for the primary data collection. For quantitative data collection, we used a pretested structured questionnaire having 13 questions divided into three parts—(1) sociodemographic information of the respondents (gender, age, education, and occupation), (2) psychological (stress, level of stress, and panic), and (3) livelihood impact (going outside for job, receiving relief and frequency, help from others, impacts on livelihood, and level of impact). Instead of income, we used occupation as a proxy to identify lower income people because people may not disclose their income data. For assessing the level of stress and level of livelihood impact, we have applied a 4-point Likert-type scale (1 = little, 2 = moderate, 3 = high, and 4 = extreme). The questionnaire was prepared in English and then translated into *Bangla*, the national language of Bangladesh. We approached respondents randomly and those who (18 years and over) agreed were interviewed. We briefly described the aims of the research to the selected respondents and their verbal consent was noted. The response rate was approximately 75%. On an average, each interview took about 15 minutes. Interviews were held at road sides, premises of small bazars, agri-farms, and tea stalls. A total of 576 respondents were interviewed in several districts of the country from March 30, 2020, to May 17, 2020. A sample size of 576 was found satisfactory at 95% confidence level and ±5% margin of error.^20^ We conducted 30 in-depth interviews randomly chosen from aforementioned samples for qualitative data gathering. A check-list consisting of a few open-ended questions was prepared to facilitate the interviews. The questions were related to the mental stress and the livelihoods of the respondents. The ethical review committee of Chittagong Medical College, Bangladesh, approved this research (Memo No. CMC/PG/2020/96).

### Data Analysis

In analyzing data, descriptive statistics (frequency and percentage) of responses were estimated. Scores of the level of livelihood impact and level of stress were the means of a 4-point Likert-type scale used. The mean difference between/among the categories of different sociodemographic characteristics with scores of the level livelihood and anxiety were compared with independent samples *t* test and one-way analysis of variance/*F* test. Besides, associations between different attributes of livelihood and anxiety with different sociodemographic characteristics were shown using the χ^2^ test. Furthermore, logistic regressions were run taking significant predictors in the χ^2^ test. Variables with more than 2 classes have been grouped into 2 categories for regressions (Supplementary Table 1, available online). Contrarily, qualitative data were described using respondents’ narratives.

## Results

### Sociodemographic Characteristics of the Respondents

The majority of respondents were men (54.7%) and aged 18 to 50 years (82.8%; Table 1). Most of them (33.3%) had no formal education and 31.4% were within primary level of education, which is the general feature of the education level of the poor people in Bangladesh. Moreover, the characteristics of lower income people of Bangladesh are also evident from respondents’ occupation status. A big portion of them (unemployed and homemakers) had no income. About 21% of them were daily workers and another 23.3% had small businesses, such as small grocery shops and tea stalls.

**Table 1.** Sociodemographic Profile of the Respondents (N = 576).

Categories	Groups	Frequency	Percentage
Gender	Men	315	54.7
	Women	261	45.3
Age group (years)	18-30	177	30.7
	31-40	145	25.2
	41-50	155	26.9
	Above 50	99	17.2
Education	No formal education	192	33.3
	Primary	181	31.4
	Secondary	122	21.2
	Diploma	81	14.1
Occupation	Unemployed	64	11.1
	Daily worker	120	20.8
	Working in industry	68	11.8
	Farmers	46	8.0
	Small business	134	23.3
	Home makers	144	25.0

### Impact of COVID-19 on Livelihoods of Lower Income People

The government of Bangladesh imposed statewide general holidays and lockdown from the middle of March 2020, which had a negative impact on the livelihoods of the general public, especially on lower income people who usually have a hand to mouth existence. This study found that 94.1% of the respondents’ livelihood was affected by the COVID-19 outbreak (Table 2). To 83% of respondents, the level of livelihood impact was high to extreme. This level of impact indicates that the lower income people were getting more marginalized than before and were becoming a member of hardcore poor due to COVID-19. A 29-year-old housemaid stated,

I live in a slum. Me and my rickshaw-puller husband are jobless now. We were not prepared for this situation at all. We have borrowed some money from others. We cannot think about our future and are worried about this so much. How can we survive if the situation goes on like this? Our neighbors are also in the same condition; everyone is tensed about their livelihood. Coronavirus made us poorer and destroyed all of our future.

As the country’s lockdown had continued for about nearly 3 months, many poor people were worried about how they would manage their family expenses. A day-laborer (30 years old) said,

Day laborers are mostly affected. I could earn 200-300 Taka (1 USD = 82 Taka) daily before the coronavirus outbreak, but now I have no income due to the lockdown situation, already sold some of our domestic animals and took loan from a local NGO for survival. We cannot take much food in our family meals; we have no option but die from hunger if this condition remains for a few more months.

The score (mean score of a 4-point Likert-type scale) for the level of livelihood impact was significantly different across sociodemographic variables (Table 3). An overall livelihood score of 3.20 ± 0.77 indicates that the livelihood impact of COVID-19 on respondents was high to extreme. This score was significantly different (*P* < .01) between/among gender, age, and occupation. However, the results of logistic regression reveal that level of livelihood impact was prominent in occupation groups (Table 4). Regarding unemployed respondents, daily workers were hardly affected by the COVID-19 outbreak (odds ratio [OR] = 7.957; 95% confidence interval [CI] = 3.551-17.834; *P* < .01), which was supported by their qualitative narratives stated above. Next to daily workers were farmers (OR = 3.91, 95% CI = 1.443-10.595; *P* < .01), industry workers (OR = 2.845, 95% CI = 1.120-7.225; *P* < .05), and homemakers (OR = 2.533, 95% CI = 1.161-2.229; *P* < .05). On the other hand, respondents aged between 31 and 40 years were more concerned (OR = 2.352, 95% CI = 1.376-4.020, *P* < .01) about the level of livelihood impact in comparison to those 18 to 30 years old. This is likely that people in this age group are the principal earning members in Bangladesh.

**Table 2.** Psychological and Livelihood Impact Issues of the Respondents (N = 576).

Variables	Response	Frequency	Percentage
Do you go outside for work during holidays?	Yes	329	57.1
	No	97	16.8
	Sometimes	150	26.0
Do you receive any relief from government during general holidays/lockdown?	Yes	163	28.3
	No	306	53.1
	Sometimes	107	18.6
If above answer is YES, then how often do you receive relief?	Only once so far	116	46.2
	Once a week	31	11.8
	Once every 2 weeks	123	42.0
Do you get help from anyone else?	Yes	170	29.5
	No	406	70.5
Is your livelihood being affected by COVID-19?	Yes	542	94.1
	No	34	5.9
If YES, at what level?	Little	13	2.3
	Moderate	85	14.8
	High	252	43.8
	Extreme	226	39.2
Do you feel stressed thinking about the outbreak of COVID-19?	Yes	441	76.6
	No	79	13.7
	May be	56	9.7
If above answer is YES, then please rate your level of stress	Little	5	1.1
	Moderate	44	10.0
	High	175	39.7
	Extreme	217	49.2
Do you think that your life become panic due to COVID-19?	Yes	426	74.0
	No	110	19.1
	May be	40	6.9

Abbreviation: COVID-19, coronavirus disease 2019.

**Table 3.** Sociodemographic Characteristics, and Score of Level of Livelihood and Psychological Impact.

Demographic variables		Level of livelihood impact^a^ ($\bar{x}\pm s$)	*t/F* test	*P*	Level of stress^b^ ($\bar{x}\pm s$)	*t/F* test	*P*
Gender	Women	3.31 ± 0.70	3.169	.002	3.19 ± 0.77	−0.137	.891
	Men	3.11 ± 0.81			3.20 ± 0.85		
Age	18-30	2.93 ± 0.80	12.087	.000	3.22 ± 0.84	4.880	.002
	31-40	3.37 ± 0.71			3.19 ± 0.77		
	41-50	3.25 ± 0.77			3.33 ± 0.73		
	Above 50	3.36 ± 0.66			2.94 ± 0.88		
Education	No formal education	3.28 ± 0.66	2.599	.051	3.17 ± 0.82	0.962	.410
	Primary	3.23 ± 0.83			3.14 ± 0.83		
	Secondary	3.17 ± 0.86			3.29 ± 0.84		
	Diploma	3.00 ± 0.69			3.23 ± 0.70		
Occupation	Unemployed	3.05 ± 0.58	9.358	.000	3.08 ± 0.77	2.89	.014
	Daily worker	3.55 ± 0.68			3.25 ± 0.83		
	Working in industry	3.21 ± 0.74			3.52 ± 0.76		
	Farmers	3.30 ± 0.78			3.21 ± 0.95		
	Small business	2.94 ± 0.83			3.10 ± 0.77		
	Home makers	3.18 ± 0.75			3.13 ± 0.81		

a Overall score livelihood impact = 3.20 ± 0.77.

b Overall score level of stress = 3.19 ± 0.81.

**Table 4.** Logistic Regression Analysis of Different Variables.

Sociodemographic variable		Odds ratio (95% confidence interval)							
		Go outside	Receive any relief	Frequency of getting relief	Get help from anyone else	Level of livelihood	Feeling stressed	Level of stress	Panic
Gender	Women								
	Men	2.322 (1.099-4.907)**	0.311 (0.164-0.589)***	0.209 (0.083-0.527)***	1.151 (0.633-2.092)	0.834 (0.480-1.450)	0.987 (0.541-1.801)		0.781 (0.426-1.431)
Age, years	18-30								
	31-40	1.077 (0.566-2.050)	0.714 (0.400-1.272)	1.829 (0.700-4.783)	0.437 (0.249-0.768)***	2.352 (1.376-4.020)***	0.639 (0.352-1.161)	5.116 (1.813-14.435)***	2.318 (1.217-4.416)**
	41-50	2.248 (1.084-4.661)**	0.773 (0.440-1.360)	2.859 (1.168-6.997)**	0.444 (0.259-0.763)***	1.721 (1.015-2.917)**	0.634 (0.345-1.167)	3.226 (1.342-7.756)***	0.810 (0.464-1.411)
	Above 50	0.934 (0.435-2.009)	0.766 (0.402-1.459)	6.321 (2.081-19.204)***	0.339 (0.172-0.668)***	1.854 (1.010-3.402)**	0.232 (0.119-0.453)***	0.994 (0.430-2.301)	0.360 (0.191-0.680)***
Education	No formal education								
	Primary	1.379 (0.681-2.791)	1.116 (0.669-1.863)	4.486 (2.180-9.233)***	2.090 (1.203-3.632)***	1.111 (0.682-1.810)	0.538 (0.311-0.930)**		1.083 (0.624-1.881)
	Secondary	0.727 (0.328-1.609)	0.302 (0.150-0.608)***	1.179 (0.364-3.817)	1.554 (0.794-3.043)	1.942 (1.037-3.637)**	0.348 (0.172-0.705)***		0.352 (0.179-0.695)***
	Diploma	0.492 (0.211-1.148)	0.256 (0.108-0.607)***	2.547 (0.513-12.647)	1.217 (0.547-2.707)	1.072 (0.493-2.332)	0.559 (0.243-1.286)		0.700 (0.309-1.586)
Occupation	Unemployed								
	Daily worker	9.984 (3.542-28.137)***	7.283 (3.270-16.217)***	2.941 (0.812-10.655)	2.113 (0.830-5.379)	7.957 (3.551-17.834)***	2.651 (1.200-5.854)**	0.511 (0.148-1.761)	0.943 (0.405-2.194)
	Working in industry	3.344 (1.243-8.999)**	13.663 (5.191-35.961)***	1.886 (0.384-9.268)	1.458 (0.519-4.100)	2.845 (1.120-7.225)**	5.818 (2.079-16.279)***	1.897 (0.426-8.452)	8.155 (2.459-27.044)***
	Farmers	7.450 (1.474-37.666)**	2.919 (1.077-7.913)**	3.277 (0.574-18.725)	1.222 (0.364-4.097)	3.910 (1.443-10.595)***	3.029 (1.079-8.502)**	0.577 (0.130-2.553)	0.649 (0.235-1.788)
	Small business	2.692 (1.210-5.988)**	0.674 (0.284-1.596)	8.857 (1.618-48.497)**	6.777 (2.773-16.566)***	1.761 (0.755-4.104)	3.440 (1.533-7.716)	0.960 (0.284-3.245)	1.222 (0.539-2.769)
	Home makers	2.943 (1.411-6.137)***	0.974 (0.489-1.941)	2.639 (0.693-10.049)	3.149 (1.276-7.770)**	2.533 (1.161-5.529)**	0.994 (0.486-2.032)	1.226 (0.346-4.346)	0.626 (0.281-1.396)

**Significant 0.05 level; ***Significant 0.01 level.

### Lockdown and Peoples’ Livelihood

Although the government declared general holidays and lockdown asking the public to stay at home in order to control the spread of COVID-19, needy people could not follow this restriction. As Bangladesh is a poverty-stricken country, the life of the poor was vulnerable due to the lockdown situation. Few days after general holidays/lockdown, the public started to move outside in search of means of livelihood. We found that 57% respondents were going outside everyday for jobs and another 26% went sometimes (Table 2). They had no other options except going outside for their livelihood. Only 17% of respondents reported staying at home due to high fear of virus infection. A daily earner (48 years old) mentioned:

For the last 2 months, I have no income. Like me, the daily earners and lower middle-class families are suffering the most because they have limited choices for their life and livelihood, and they are facing many difficulties nowadays. So, many people are compelled to go outside to earn a minimum income. An empty stomach does not listen to anything; the poor have to move outside as they do not have food in their houses.

Logistic regressions (Table 4) show that daily workers were going outside more frequently in comparison to unemployed respondents (OR = 9.984, 95% CI = 3.542-28.137, *P* < .01) followed by farmers (OR = 7.450, 95% CI = 1.474-37.666, *P* < .05) and respondents working in industries (OR = 3.344, 95% CI = 1.243-8.999, *P* < 0.05). On the other hand, men (OR = 2.322, 95% CI = 1.099-4.907, *P* < .05) tended to go outside more than women, and 41- to 50-year-old respondents (OR = 2.248, 95% CI = 1.084-4.661, *P* < .05) went outside more frequently than 18- to 30-year-old respondents.

### Relief and Aid for Poor People During Lockdown

To lessen the financial burden, the government provided assistance in terms of both kind (eg rice, oil, sugar, etc) and cash to the needy public in the country. However, our findings show that 53% of the respondents did not receive any such assistance during the COVID-19 outbreak (Table 2). Only 28.3% of the respondents said “yes” and another 18.6% received assistance sometimes. Among those who received the government’s relief, only 11.8% of them received once in a week and 46.2% received just once. Another 42% of them received once in every 2 weeks. This means that respondents did not receive the government’s assistance regularly. However, 29.5% of the respondents reported that they got help from other agencies (nongovernmental organizations) and individuals. A 39-year-old farmer reported:

I received government relief once in the last 2 months through our area representative. We need more support as we depend on our everyday earning. I don’t have any savings, so I expect more aid from different people including the government.

As expected, respondents working in industries (OR = 13.663, 95% CI = 5.191-35.961, *P* < .01), daily workers (OR = 7.283, 95% CI = 3.270-16.217, *P* < .01), and farmers (OR = 2.919, 95% CI = 1.077-7.913, *P* < .05) received more assistance than unemployed respondents (Table 4). Respondents over 50 years old received more frequent assistance compared with 18- to 30-year-old respondents. Surprisingly, respondents having small business received government’s relief frequently (OR = 8.857, 95% CI = 1.618-48.497, *P* < .01) as well as help from others (OR = 6.777, 95% CI = 2.773-16.566, *P* < .01).

### Psychological Impact of COVID-19 Outbreak

Three attributes (eg stress, level of stress, and panic) were evaluated to understand the psychological impact of COVID-19 on lower income people (Table 2). The majority of the respondents (76.6%) said that they were feeling stressed due to the outbreak of COVID-19 and another 9.7% were not sure of COVID-19’s stress. Of those who reported they were stressed, 89.9% of them were high to extremely stressful and only 1.1% of them reported having little stress. From qualitative interviews, we understood that many of them lost their jobs and income due to lockdown, and the borrowed loan from relatives, friends, or others ultimately made them stressed. A garment worker (39 years old) expressed her feelings in the following way:

We are suffering from different mental stresses as we have no cash in hand; don’t know how we will face the future; cannot even sleep properly at night. It is better to die of coronavirus than hunger. Most people cannot take a proper meal, they eat only once daily. We are scared of our future; if the situation continues, we would not survive. Most poor (people) have been passing a horrible time as most of them borrowed money to manage their everyday meals. Debt is increasing. Only God knows what will happen.

The overall score of the level of stress which was 3.19 ± 0.81 indicates that the respondents were high to extremely stressful (Table 3). The score of the level of stress was significantly different among respondents of different age and occupation groups. Respondents working in industries and daily laborers were found to be more stressed. Similarly, higher scores were noticed for respondents within 31 to 50 years old who are the earning members of families. A day laborer (55 years old) mentioned:

The poor people are the worst sufferers as they have become jobless. I am worried too much. There is no end to our sorrows. I used to earn daily before the coronavirus outbreak, but now I do not have a regular income. As the disease has been spreading widely, will I be able to do my work in the coming days?

Fear of COVID-19 infection, lack of jobs and income, all these had made respondents’ life panic stricken. More than 70% of the respondents reported to be in panic. A slum dweller woman (33 years old) expressed her feelings:

Corona is the name of a fear. People are dying in different countries from this virus. This is a curse from God. In the slum, poor people live in a small house; have no way to maintain social distancing, washing hands again and again, and using gloves. We will be the most infected person as we are poor. We have very limited access to a doctor or hospital because of our financial constraints.

The results of logistic regressions also support the above psychological impact of COVID-19. Respondents working in industries (OR = 5.818, 95% CI = 2.079-16.279, *P* < .01), farmers (OR = 3.029, 95% CI = 1.079-8.502, *P* < .05), and day laborers (OR = 2.651, 95% CI = 1.200-5.854, *P* < .05) were feeling higher stress in comparison to unemployed respondents (Table 4). The level of stress was higher in respondents within 31 to 40 years (OR = 5.116, 95% CI = 1.813-14.435, *P* < .01) and 41 to 50 years age range (OR = 3.226, 95% CI = 1.342-7.756, *P* < .01) in comparison to 18- to 30-year-old respondents. These attributes were also reflected in respondents’ panic condition. Those respondents who worked in industries were more panicked than unemployed (OR = 8.155, 95% CI = 2.459-27.044, *P* < .01).

## Discussion

This research looked at the impact of the COVID-19 outbreak on the livelihood and the psychological stress of lower income people of suburban Bangladesh. An overwhelming percentage of the respondents reported that due to COVID-19’s lockdown, it was hard for them to find a job to maintain the daily family expenses. The effects of COVID-19 pandemic are not only limited to health but also have a major impact on the social and economic aspects.^21^ Reports expressed that the COVID-19 would badly affect the livelihood of almost half of the global workforce both in cities and rural areas.^22,23^ It was reported that more than 10 million people will be further marginalized due to the loss of wages and jobs in Bangladesh.^24^ Bodrud-Doza et al reported that many low- and middle-income people of Bangladesh will lose their jobs and income sources due to COVID-19 outbreaks.^16^ In the past outbreaks also, it was noticed that the poor and underprivileged people were seen to suffer the most from the prevalence of any infectious disease.^25,26^

In order to support the socioeconomic situation of the country during the COVID-19 outbreak, the government had declared stimulus packages and social safety net programs. However, the economic stimulus programs were designed for large industries and service sectors.^27^ Nonetheless, on a priority basis, the financial incentives should be given to the poverty-stricken disadvantaged communities first.^16^ Our study found that only a small fraction of the respondents received government support occasionally. Shammi et al reported that the ultra-poor are often being left out of the relief program during the disasters.^11^ A sense of governance from public service providers is indispensable to ensure that benefits from the government’s social safety net programs reach needy people. This is also important, during this unusual period, to think of alternative small-scale enterprises where people can take part from home and earn a minimum income for their subsistence. Relevant authorities, both government and private, can come forward to initiate/support such endeavors. If these happen, public movement would be reduced and community transmission of COVID-19 could be controlled. Due to the opening of economic activities and uncontrolled movement of people, positive cases of COVID-19 in Bangladesh have still remained more than a thousand every day (1320 confirmed cases on November 2, 2020), though the daily reported cases were more than 3000 during June-July of the same year.^14^

Limited economic opportunities, restricted movement, less scope of meeting relatives, friends, and neighbors from whom they used to seek help in emergencies, fear of being infected by COVID-19, and lack of confirmed support from the government—all had made lower income peoples’ life vulnerable and stressful. A large percentage of the respondents of this study were worried about their survival due to the absence of regular income. They were struggling to maintain their personal hygiene because of financial constraints and so became alarmed with the fear of being infected by COVID-19. In any epidemic and emergencies, it is common for individuals to feel stressed, panicked, and weakened social networks.^8,9^ Rajkumar highlighted the need for mental health services, particularly for vulnerable populations, and the strengthening of social networks to reduce the adverse psychological impact of any outbreak.^10^ The mental health and well-being of whole societies have been severely affected by the COVID-19 crisis and are a priority to be addressed urgently.^8^ Adoption of a “Whole-of-Society” approach in COVID-19 national response to promote, protect, and care for mental well-being is essential because it improves the quality of programming, enhances coping skills of the public during the crisis, reduces suffering, and is likely to speed up the recovery and rebuilding of communities.^8,9^

## Conclusions

The COVID-19 pandemic has brought huge socioeconomic and psychological sufferings in human life all over the world. Even though all groups of people in society have been affected, the lower income people have become more vulnerable. In developing countries, where the majority of the people depend on their daily income, the COVID-19 outbreak has threatened their subsistence. The absence of livelihood means, fear of getting infected by coronavirus, and insufficient government’s assistance has made their livelihood vulnerable and life stressful. In order to contain the coronavirus transmission, the public movement needs to be controlled, which requires measures such as lockdown, isolation, and social distancing. For effective implementation of these measures in a densely populated country like Bangladesh, underprivileged people should be provided with essential government assistance during a particular period. Otherwise, disease transmission would prolong and this might have a long-term impact on the country. This research was an initiative to quickly discern the impact of COVID-19 on the psychological condition and livelihood of lower income people in Bangladesh. This research had some limitations. First, it interviewed only 576 people in several districts of Bangladesh. So, it may not be reasonable to generalize the results in the whole country. Further studies can be conducted with more participants distributed throughout the country. Second, we considered only a limited number of variables to understand the impact. However, future studies can accommodate more variables of psychological and livelihood impact so that a general scenario of the impact can be produced extensively.

## Supplemental Material

sj-pdf-1-aph-10.1177_1010539520977304 – Supplemental material for Psychological and Livelihood Impacts of COVID-19 on Bangladeshi Lower Income People Click here for additional data file. Supplemental material, sj-pdf-1-aph-10.1177_1010539520977304 for Psychological and Livelihood Impacts of COVID-19 on Bangladeshi Lower Income People by Alak Paul, Tapan Kumar Nath, Janardan Mahanta, Naznin Nahar Sultana, A. S. M. Imrul Kayes, Sharifa Jahan Noon, Md. Akib Jabed, Sumana Podder and Sujat Paul in Asia Pacific Journal of Public Health
