# Supplementary material for: Psychological and Livelihood Impacts of COVID-19 on Bangladeshi Lower Income People
Source: Asia Pac J Public Health. 2020 Dec 8;33(1):100–8. doi: 10.1177/1010539520977304 (PMC7934155; doi:10.1177/1010539520977304)
Supplement: sj-pdf-1-aph-10.1177_1010539520977304 – Supplemental material for Psychological and Livelihood Impacts of COVID-19 on Bangladeshi Lower Income People [file sj-pdf-1-aph-10.1177_1010539520977304.pdf]

Supplementary Table 1. Category of selected variables for logistic regression

Go outside during holidays				Binary for Logistic Regression	
Yes	No	Sometimes		No (0)	Others (1)
Receive any relief					
Yes	No	Sometimes		No (0)	Others (1)
Frequency of getting relief					
Only once	Once a week	two weeks		Only once (0)	Others (1)
Get help from anyone else					
Yes	No			No (0)	Yes (1)
Level of livelihood					
Little	Moderate	High	Extreme	Others (0)	Extreme (1)
Feeling anxious about covid-19					
Yes	No	Maybe		Others (0)	Yes (1)
Level of anxious					
Little	Moderate	High	Extreme	Little & Moderate (0)	High & Extreme (1)
Panic about COVID-19					
Yes	No	Maybe		Others (0)	Yes (1)
Level of livelihood					
Little	Moderate	High	Extreme	Others (0)	Extreme (1)
